# Choice of population structure informative principal components for adjustment in a case-control study

**DOI:** 10.1186/1471-2156-12-64

**Published:** 2011-07-19

**Authors:** Gina M Peloso, Kathryn L Lunetta

**Affiliations:** 1 Department of Biostatistics, Boston University School of Public Health, 801 Massachusetts Avenue, Boston MA, 02118, USA

## Abstract

**Background:**

There are many ways to perform adjustment for population structure. It remains unclear what the optimal approach is and whether the optimal approach varies by the type of samples and substructure present. The simplest and most straightforward approach is to adjust for the continuous principal components (PCs) that capture ancestry. Through simulation, we explored the issue of which ancestry informative PCs should be adjusted for in an association model to control for the confounding nature of population structure while maintaining maximum power. A thorough examination of selecting PCs for adjustment in a case-control study across the possible structure scenarios that could occur in a genome-wide association study has not been previously reported.

**Results:**

We found that when the SNP and phenotype frequencies do not vary over the sub-populations, all methods of selection provided similar power and appropriate Type I error for association. When the SNP is not structured and the phenotype has large structure, then selection methods that do not select PCs for inclusion as covariates generally provide the most power. When there is a structured SNP and a non-structured phenotype, selection methods that include PCs in the model have greater power. When both the SNP and the phenotype are structured, all methods of selection have similar power.

**Conclusions:**

Standard practice is to include a fixed number of PCs in genome-wide association studies. Based on our findings, we conclude that if power is not a concern, then selecting the same set of top PCs for adjustment for all SNPs in logistic regression is a strategy that achieves appropriate Type I error. However, standard practice is not optimal in all scenarios and to optimize power for structured SNPs in the presence of unstructured phenotypes, PCs that are associated with the tested SNP should be included in the logistic model.

## Background

The principal components (PCs) of genome-wide genotype data can be used to detect and adjust for population structure in genetic association analyses [1,2]. The popularity of the PC method is evident by its wide use: it has been cited by over 400 publications. However, the choice of which PCs to use and the best way to adjust for the PCs in analyses of dichotomous traits is not yet clear.

Numerous methods have been proposed to adjust for structure once PCs are computed (Table 1). The simplest and most straightforward approach is to adjust for continuous PCs in a regression model. Kimmel et al [3] note that principal component analysis (PCA) is sufficient for identifying population structure, but adjusting for PCs as covariates in a model may not always eliminate false positive associations since the PCs are only an estimate of the population structure. Furthermore, Yu et al [4] show that adjusting for unnecessary PCs can have a negative impact on power in case-control studies when the PCs are distributed equally in cases and controls or ancestry has already been accounted for by other variables in the model. Including PCs that do not account for structure in the model adds noise and therefore reduces the power to find a true effect. Genetic population structure depends on the genotypes, unlike typical covariates, such as age and sex, which are not dependent on the candidate genotypes. Since the PCs are linear combinations of genome-wide genotypes, the behaviour of association models that include both SNP genotypes and PCs warrants further investigation.

**Table 1:** Methods of ancestry informative PC selection.

Method	First Author
Adjusting for a fixed number of PCs	Price[2]

Tracy-Wisdom statistic	Patterson[1]

Regression of outcome on PCs	Novembre[23], Peloso[20]

Reduction in inflation of genomic control lambda	Yu[4]

PC-Finder	Li[21]

10% rule	Jewell[24]

PCs + cluster	Li[25]

Optimal criteria for selecting PCs to include in the model are not known. Suppose the population from which we draw our sample consists of two sub-populations. We can expect one of four scenarios (Table 2). A structured phenotype indicates different probabilities of case status in the two sub-populations (K_1_≠K_2_), while a non-structured phenotype has equal probably of being a case in the two sub-populations (K_1 _= K_2_). Likewise, a structured SNP (sSNP) has unequal risk allele frequency in the two sub-populations (p_1_≠p_2_), and a non-structured SNP (nsSNP) has equal risk allele frequency in the two sub-populations (p_1 _= p_2_). By testing the phenotype for association with the PCs, we can determine if the phenotype is structured (scenarios A and C of Table 2) or non-structured (scenarios B and D). However, each SNP to be tested for association must be tested individually to determine structure status. Our goal was to determine the optimal PCs to use in a case/control association analysis under the 4 scenarios we can encounter across the genome. We define optimal as the adjustment that yields the greatest power while maintaining appropriate Type I error.

**Table 2:** Scenarios of population structure that could occur across the genome.

	Structured Phenotype (K_1_≠ K_2_)	Non-Structured Phenotype (K_1 _= K_2_)
**Structured Genotype** **(p_1_≠ p_2_)**	A	B

**Non-Structured Genotype** **(p_1 _= p_2_)**	C	D

p_1 _and p_2 _are the allele frequencies in population 1 and 2, respectively. K_1 _and K_2 _are the frequency of disease in population 1 and 2, respectively.

In a genetic association study, our primary interest is not in finding and describing the genetic structure in the sample, but in determining if the population structure in the sample has a confounding effect on the SNP association analyses and if adjustment for this confounding is necessary. We performed simulation studies to investigate the Type I error and power of associations between case/control status and a SNP when adjusting for PCs selected using samples of independent individuals. We compared the following methods of selecting PCs (label):

(1) No PC adjustment (None)

(2) 10% Rule (10% Rule)

(3) PCs significantly related to the outcome at significance level α = 0.001, 0.01, or 0.05 (Sig001, Sig01, Sig 05, respectively)

(4) PCs significantly related to the SNP at α = 0.05 or 0.01 (SNP01, SNP05, respectively)

(5) PCs significant according to the Tracy-Widom statistic at α = 0.05 (TW)

(6) Top PCs (2 or 10) determined according to eigenvalue (Top2, Top10, respectively)

(7) Simulated true population i.e, Gold standard (Pop)

We tested for association with the simulated case/control outcome using logistic regression and compared Type I error and power of associations between the outcome and SNP when adjusting for selected principal components of ancestry. Finally, to provide a practical example of the methods of PC selection, we performed all methods of PC selection using dichotomized height data from the Framingham Heart Study.

## Methods

We simulated independent genome-wide SNPs by generating ancestral population allele frequencies for 10,000 SNPs (p_j_, j = 1,...,10000) from a uniform (0.05, 0.50)-distribution. We then created two sub-populations (i = 1,2) of 500 individuals, each descending from the ancestral population according to F_st_. We simulated the allele frequencies p_ij _(i = 1,2; j = 1,..., 10000) in the two sub-populations according to a beta distribution [5]:

. For each individual in population i, the genotype probabilities for 0, 1, or 2 minor alleles for SNP j were assigned with probabilities:

F_st _is a measure of population differentiation [5]; F_st _= 0.01 is representative of human population structure seen within continents, while F_st _= 0.1 is representative of structure seen between continents [6,7]. We simulated our samples using F_st _values of 0.01 and 0.1. 1,000 replicates of independent genome-wide SNP data were generated for 2 populations of 500 individuals.

We combined the two generated sub-populations and performed PCA using the smartpca program in EIGENSOFT [1]. To evaluate Type I error and power for association, we simulated a test phenotype and test SNP genotype within each sub-population (Table 3). We set the frequency of disease in sub-population 1 to be between 10% and 19% and set the frequency of disease in population 2 so that the overall prevalence of disease in the combined populations was 10%. We selected a total of 500 cases, between 250 and 475 coming from sub-population 1 and the remaining from sub-population 2. Under no phenotypic population structure, the number of cases and controls in the sample from each population are equal: 250 cases and 250 controls. Under phenotypic population structure, we set the disease frequency, and therefore the proportion of cases in the sample coming from sub-population 2, to be 1.5, 3, 4, or 19-fold higher than sub-population 1 (Table 4). The controls were selected so that the sample had a total of 500 individuals from each generated sub-population. For example, for samples with 375 cases from population 1, there are 125 controls from population 1. The risk allele frequency in sub-population 1 was set to 10, 20, or 30%, and the risk allele frequency in sub-population 2 was set so that the overall risk allele frequency in the combined populations was 20%. When the test SNP allele frequency is set to be the same in the two sub-populations, there is no genotypic structure. To simulate a genetic effect of the test SNP on disease, we simulated an odds ratio of 1 for evaluating Type I error; for power we simulated under a log-additive model with odds ratios of 1.2 or 1.5.

**Table 3:** Simulation parameters.

Description	Possible Values
Population differentiation (F_st_)	0.01, 0.1
Population prevalence of disease (K)	0.10
Frequency of disease in sub population 1 (K_1_)	0.10 -- 0.19
Number of cases in sub-population 1	250 - 475
Overall risk allele frequency (p)	0.20
Risk allele freq in sub-population 1 (p_1_)	0.10 -- 0.30
Odds Ratio (log additive model)	1.0, 1.2, 1.5

**Table 4:** Relationship between number of cases and population prevalence of disease in each sub-population.

# of Cases in Population 1	# of Cases in Population 2	Fold increase in # of cases	K_1_	K_2_
250	250	1	0.10	0.10
300	200	1.5	0.12	0.08
375	125	3	0.15	0.05
400	100	4	0.16	0.04
475	25	19	0.19	0.01

We compared the methods for selecting PCs for adjustment described above. We used logistic regression to test the association between the test SNP and case status adjusting for latent ancestry defined by the PCs. We compared the proportion of replicates significant at α = 0.05, using 1,000 replicates for each set of parameters to investigate Type I error and power. 1,000 replicates for Type I error provides a 95% confidence interval of 0.036 to 0.064 around a nominal significance level of 0.05.

To determine the effects of the PC selection methods when a sample is composed of a more complex structure, we simulated two populations that each diverged with as F_st _of 0.01 from an ancestral population, as previously. We treated these two subpopulations as ancestral populations and then simulated two subpopulations diverging from each of the ancestral populations, again with an F_st _of 0.01. The resulting sample had four sub-populations. Due to computational limitations, a single replicate of independent genome-wide SNP data was generated for this scenario for PCA. As before, 1,000 replicates were used to evaluate Type I error and power, simulating the genotype and phenotype (conditional on genotype for power) for each replicate. We varied the phenotypic and genotypic structure of the sub-populations, from having no structure to more extreme structure.

## Results and Discussion

### Simulation Study

**Figure 1:**
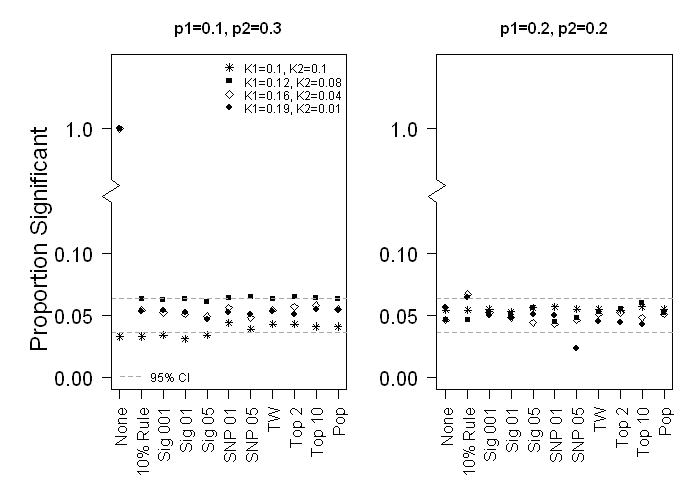
**Empirical Type I error results**. Two sub-populations of 500 individuals each, F_st _= 0.01. K_1 _and p_1 _are the population prevalence of disease and risk allele frequency, respectively, in sub-population 1. K_2 _and p_2 _are the population prevalence of disease and risk allele frequency, respectively, in sub-population 2. The x-axis is the various methods of selecting PCs for inclusion in the model of association and the symbols in the plot represent the phenotypic structure. The y-axis is the proportion of logistic regression models adjusting for the selected PCs for which the SNP p-values are significant at a significance level of 0.05.

Type I error for the methods of selection with two subpopulations with F_st _of 0.01 is provided in Figure 1. The 95% confidence band in the plots is the 95% confidence interval around 0.05. Type I error is at the nominal level or conservative as long as a PC selection method is used. As expected, when no adjustment is made for population structure, we found that appropriate Type I error is observed only as long as either the SNP or the outcome is not structured. If both the SNP and the outcome are structured, as is well known, there is highly inflated Type I error when no PCs are included as covariates. Also as expected, we did not observe inflated Type I error rates with a structured SNP and a non-structured phenotype, nor with a non-structured SNP and a structured phenotype.

**Figure 2:**
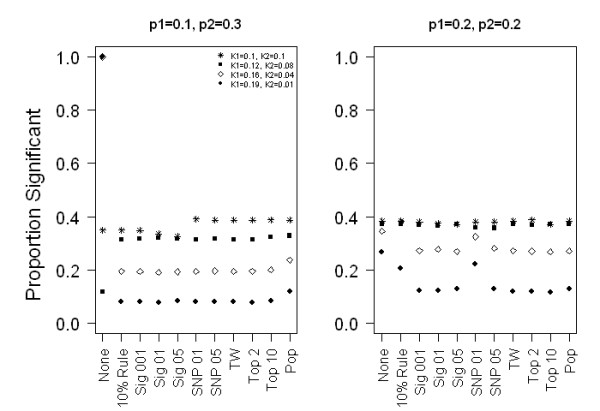
**Empirical power results**. Two sub-populations of 500 individuals each, F_st _= 0.01, simulated log additive odds ratio of 1.2. K_1 _and p_1 _are the population prevalence of disease and risk allele frequency, respectively, in sub-population 1. K_2 _and p_2 _are the population prevalence of disease and risk allele frequency, respectively, in sub-population 2. The x-axis is the various methods of selecting PCs for inclusion in the model of association and the symbols in the plot represent the phenotypic structure. The y-axis is the proportion of logistic regression models adjusting for the selected PCs for which the SNP p-values are significant at a significance level of 0.05.

Figure 2 provides power estimates with a simulated odds ratio of 1.2. When the phenotypic frequency does not vary between the subpopulations (a non-structured phenotype) but the frequency of the SNP does (structured SNP), we found that logistic models that include PC covariates (Top2, Top10, TW, SNP01, SNP05) have the highest power. When both the phenotype and SNP are structured, the power is similar for all PC selection methods, since all appropriately select at least one PC for adjustment. However, when the phenotypic frequency differs substantially between sub-populations but the SNP is not structured, power is lower for selection methods that include PCs in the model compared to those that do not. Therefore, to obtain optimal power for a non-structured SNP and substantially structured phenotype, selection methods that would not include PCs in the model are best; in this case, methods that select PCs based on their association with the SNP (SNP01), and methods that select PCs based on association with phenotype perform less well (Sig001, Sig01, Sig05, Top2, Top10). We found similar patterns with a simulated odds ratio of 1.5, and when the F_st _between the sub-populations was 0.1.

We next expanded this simulation to larger differences in allele frequencies between the two sub-populations. With large F_st _(0.10), we expect many SNPs with greater than a 0.2 difference in allele frequency between sub-populations, and thus we believe it may be more common to observe large allele frequency differences between populations than large phenotypic differences between populations. We found that as the difference in the risk allele frequency between the two sub-populations increases, the difference in power between adjusting and not adjusting for PCs becomes greater (see additional file 1) when a non-structured phenotype and structured SNP were tested for association. Consistent with our previous results, we found when both the phenotype and the SNP are structured any method of selection was adequate. When the SNP is non-structured (p_1 _= p_2 _= 0.5) and the phenotype has large structure (phenotypic ratio = 4), we found slightly higher power when selecting PCs by the 10% rule compared to not selected any PCs, contrary to the findings presented in Figure 2. As seen in additional file 1, the difference in Type I error between using the 10% rule method versus no PC selection method is similar to the difference in power. The difference between the two analyses is that PC1 is included in 16% of the replicates for the 10% rule.

Finally, we increased the sample size for the genome-wide SNPs simulation to 5,000 individuals from each sub-population. Increasing the sample size allowed us to determine if our observed results were affected by the simulated sample size of 500 cases and 500 controls. We found the same patterns with the larger sample size as we did when we used the 500 individuals from each sub-population (results not shown).

In general, we observed similar patterns when the data consisted of four sub-populations as with the two sub-populations scenario already presented (see additional file 2). When the SNP and phenotype frequencies do not vary over the sub-populations, all methods of selection provided similar power and appropriate Type I error for association. When the SNP is not structured and the phenotype has large structure, then selection methods that do not select PCs for inclusion as covariates provide the most power. Likewise, when there is a structured SNP and a non-structured phenotype, selection methods that include PCs in the model have greater power. When both the SNP and the phenotype are structured, all methods of selection have similar power, except when the SNP differs between the 4 sub-populations and only the top 2 PCs are selected for adjustment. In this case, we observe elevated Type I error and loss of power because three PCs are required to distinguish 4 subpopulations, and only 2 PCs are included in the model. The loss of power when we do not adjust fully for the population structure is due to an attenuation of the effect due to the population structure (negative confounding). Positive confounding occurs when the structure is in the same direction as the true genetic effect, or that the phenotypic means and risk allele frequencies are positively correlated. Negative confounding occurs when the structure is in the opposite direction as the true genetic effect, or in other words, that the phenotypic means and risk allele frequencies are negatively correlated [8]. While we only report results here based on negative confounding, simulations with positive confounding yielded similar conclusions (see additional file 3).

Overall, we find that for some scenarios, the optimal choice of PCs to adjust for in a genome-wide association study using logistic regression is SNP-dependent (Table 5). We define optimal as maximal power while maintaining appropriate Type I error. When both the SNP and the phenotype are structured or both are non-structured, then any of the methods of PC selection will maintain reasonable Type I error and have similar power. This is most likely because all of the approaches retain at least the first PC when both the SNP and the phenotype are structured. When the SNP is not structured but the phenotype has substantial structure (K_1 _= 0.19, K_2 _= 0.01), selection methods that result in no PC adjustment (SNP01, 10% Rule, None) have optimal power. Conversely, when the phenotype is not structured and the SNP is structured, we achieve optimal power when PCs are included in the model, e.g., for the selection methods that include a fixed number of PCs or PCs associated with the SNP in the model (TW, Top2, Top10, SNP01, SNP05). With logistic regression, when adjusting for non-confounding covariates (covariates only associated with the outcome) there is a loss in precision [9]. But, omitting balanced covariates (covariates that are uncorrelated with the outcome in the sample), biases the results towards the null, whereas the results are not biased if the balanced covariates are included in the model of association. In some situations, the increased precision achieved by omitting the covariates improves the power despite the bias of the parameter estimate for the SNP toward the null.

**Table 5:** Scenarios that could occur across the genome with the optimal method of selection.

	Structured Phenotype (K_1 _≠ K_2_)	Non-Structured Phenotype (K_1 _= K_2_)
**Structured Genotype** **(p_1_≠ p_2_)**	Any method of selection except no PC adjustment	Selecting a fixed number of PCs or PCs associated with the SNP

**Non-Structured Genotype** **(p_1 _= p_2_)**	Selecting PCs associated with the SNP (α = 0.01), 10% Rule, no PC adjustment	Any method of selection

p_1 _and p_2 _are the allele frequencies in population 1 and 2, respectively. K_1 _and K_2 _are the frequency of disease in population 1 and 2, respectively.

**Figure 3:**
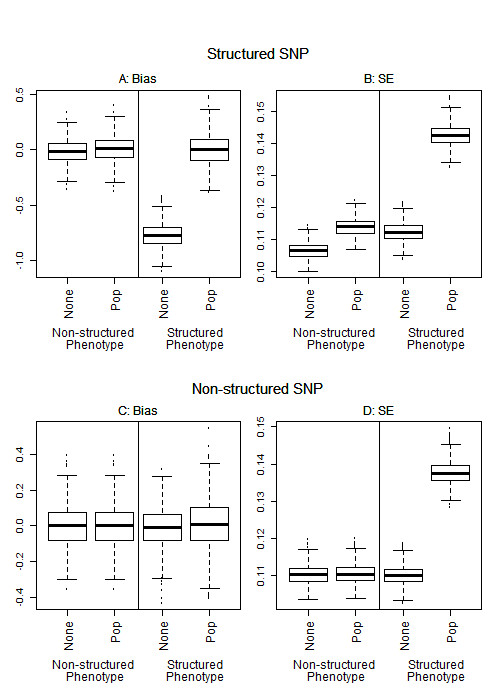
**Bias and standard error (SE)**. Two sub-populations of 500 individuals each, F_st _= 0.01, simulated log additive odds ratio of 1.2. Bias was computed as the estimated effect minus the true simulated beta. For the non-structured phenotype, each sub-population had a population prevalence of disease of 0.1. For the structured phenotype the population prevalence of disease was 0.16 in sub-population 1 and 0.04 in sub-population 2. The non-structured SNP had a frequency of 0.2 in each population, and the structured SNP had a frequency of 0.1 in sub-population 1 and 0.3 in sub-population 2. None indicates no PCs were adjusted for in the model and Pop indicates that the known population was adjusted for in the model.

We explored the bias and standard error of our models to better understand the dependency of power on the SNP structure (Figure 3). Biased estimates occur when both the phenotype and SNP are structured and no population structure covariate (PCs or true population) is included in the model (Figure 3A). There is a loss of precision (increased SE) when the phenotype is structured and the SNP is not and a population structure covariate is included in the model (Figure 3D), resulting in the decreased power. Likewise, when the SNP is structured but the phenotype is not, the SE is smaller if a population structure covariate is not included in the model (Figure 3B), but there is a negative bias when no adjustment is included, resulting in decreased power. As far as we know, no one has proposed to look at the structure of a SNP to determine whether to adjust for structure in genome-wide association studies with binary outcomes.

### Example of Principal Component Selection Criteria with Height

Average adult height is taller in northern Europe than in southern Europe. By our definition, height is a structured phenotype, i.e., it varies by ancestry. Lactose intolerance also varies across Europe from North to South. The genetic polymorphism in the LCT (Lactase) gene that causes lactose intolerance, and the SNPs in LD with this polymorphism, appears to be associated with height in non-homogeneous samples of individuals of European descent [10]. Observing an LCT-height association in a sample indicates the sample has population structure. As an example of using the various methods of PC selection in practice, we investigated the association between height and four SNPs in the Framingham Heart Study, adjusting for selected PCs. Because our interest is in dichotomous outcomes, we dichotomized height by the median for this example, and used logistic regression to test for association between four SNPs and dichotomized height:

• rs1042725 and rs6060369 [11]: Two positive control SNP not in the LCT gene that are known to be associated with height. Both SNPs are associated with PC1 with p-values of 9.67E-08 and 0.0003, respectively.

• rs2322659 [10]: A structured SNP in the lactase gene which is known to vary in frequency among European Americans. This SNP is highly associated with PC1 (p-value = 3.8E-73).

• rs2290305: a non-structured SNP, not associated with PC1 (p-value = 0.425).

Table 6 displays the results of the association between dichotomized height and the SNPs adjusting for PCs chosen by the various methods of selection. For the structured SNP (rs2322659), we found that when we do not adjust for PCs, we obtain a false positive association, but the association diminishes when PCs are included in the model. For the positive control SNPs (rs1042725 and rs6060269), we found that adjusting for PCs can either enhance or diminish the association with height. This is probably due to whether the structure is a positive or negative confounder of the association [12].

**Table 6:** Height association results with methods for selecting PCs.

		beta (p-value) of SNP			
		
		sSNP*	nSNP**	Positive Control SNPs	
**Method for selecting PCs**	**Selected PCs**	**rs2322659**	**rs2290305**	**rs1042725**	**rs6060369**

No PC Adjustment	NA	-0.406 (< 0.001)	-0.086 (0.338)	-0.251 (0.002)	0.221 (0.007)

Top 2 PCs	PC1, PC2	-0.043 (0.646)	-0.064 (0.491)	-0.149 (0.073)	0.336 (< 0.001)

Top 10 PCs	PC1 - PC10	-0.057 (0.563)	-0.054 (0.571)	-0.132 (0.121)	0.322 (< 0.001)

Tracy-Widom statistic	PC1 - PC81	-0.062 (0.566)	-0.057 (0.581)	-0.174 (0.061)	0.318 (0.001)

Associated with the outcome at α = 0.05	PC1, PC2, PC4, PC8, PC21, PC25, PC28, PC39, PC47, PC49, PC56, PC64, PC77	-0.03 (0.762)	-0.049 (0.617)	-0.164 (0.059)	0.313 (0.001)

Associated with the outcome at α = 0.01	PC1, PC4, PC21, PC25, PC28, PC49, PC77	-0.015 (0.875)	-0.053 (0.578)	-0.162 (0.059)	0.322 (< 0.001)

Associated with the outcome at α = 0.001	PC1, PC4, PC28	0.014 (0.884)	-0.047 (0.619)	-0.163 (0.055)	0.322 (< 0.001)

Associated with the SNP at α = 0.05	varied by SNP	-0.041 (0.677)	-0.092 (0.312)	-0.148 (0.075)	0.333 (< 0.001)

Associated with the SNP at α = 0.01	varied by SNP	-.038 (0.703)	-0.086 (0.338)	-0.164 (0.170)	0.313 (< 0.001)

10% Rule	PC1#	-0.037 (0.692)	-0.086 (0.338)	-0.148 (0.075)	0.333 (< 0.001)

PC-Finder [21]	NA	-0.406 (< 0.001)	-0.086 (0.338)	-0.251 (0.002)	0.221 (0.007)

Regression estimate (p-value) of the association between outcome and SNP adjusting for the selected PCs are displayed in the table. *sSNP is a structured SNP. This SNP is in the lactase gene, and varies in frequency across Europe; ** nSNP is a non-structured SNP. This SNP does not vary among subgroups in the FHS sample; # PC1 was selected except for the nSNP (rs2290305), in which case, no PCs were selected by the 10% rule.

## Conclusions

We performed a simulation study in which we generated multiple sets of genome-wide SNPs. The goal was to investigate Type I error and power of associations between case-control status and a SNP when adjusting for ancestry informative PCs selected by a variety of rules. A second aim of this study was to examine more critically the effects of the amount of phenotypic structure and genotypic structure on the association analysis, as well as investigate the bias and precision of the associations.

We did not specifically address the issue of which SNPs to include in the PCA. Using all available SNPs in a PCA provides the maximal information to ancestry, but highly correlated SNPs or unusual chromosomal phenomena such as known inversion polymorphisms or genomic regions known to play a role in susceptibility to a disease can affect the results from a PCA [13]. Under some conditions, including the chromosomal regions with high influence in PCA may have a negative impact on power when PCs are used to adjust for ancestry. For example, if the region harbours a true genetic effect, the effect may be adjusted away. Thus, some researchers have recommended not using PCs that are correlated with localized chromosomal regions [14].

All simulations were performed using distinct sub-populations. Admixed individuals are commonly used in GWAS. While we did not explicitly simulate admixed individuals, we know based on previous work [15] that PCA to detect ancestry and subsequent adjustment works similarly with admixed individuals having global phenotypic structure. On the other hand, PCs based on genome-wide data do not adequately capture local ancestry or local phenotypic structure [16,17]. If local phenotypic structure exists, other techniques need to be applied to capture and adjust for local ancestry such as PCA in the region of the test SNP, or methods that estimate local ancestry proportions such as ANCESTRYMAP [18] or LAMP [19]. Further work needs to be done to determine how to adjust for local ancestry without adjusting away true effects.

We focused our exploration on linear PC adjustment models. We did not investigate adjusting for clusters identified in the individual genotype data because previous work has suggested that linear adjustments are adequate for the population structure typical of European populations [20]. We did not investigate the method of testing the reduction in the inflation of the genomic control lambda [4] or PC-Finder [21] due to the computational burden of these algorithms. Prior to excluding these methods, we investigated the run time for the PC-Finder algorithm and found that it is related to the number of PCs selected. When more PCs are needed to adjust for the structure, the algorithm takes longer to run. With simulated genotypes similar to those presented in this chapter, PC-Finder requires between 5 minutes and one hour to select PCs. While these methods may be feasible in individual data sets where the algorithm needs to be run only once for the outcome of interest, it is not suited for simulations requiring thousands of replicates. Furthermore, we found in our dichotomized height example that PC-Finder did not select any PCs for adjustment and therefore did not remove the false association with the LCT SNP.

Our findings suggest that to optimize power under certain scenarios, the choice of covariate PCs in a genome-wide association study using logistic regression with a dichotomous outcome should be SNP-dependent. Our findings only apply to case-control or dichotomous outcome analyses using logistic regression. These results may appear to conflict with Xing and Xing [22], who recently clarified that covariate adjustment in logistic regression always leads to a loss of precision, but not always a loss of power. They conclude that when the genotype and covariates are independent, it is still more efficient to adjust for the predictive covariates. In contrast, we found that with large phenotypic structure and non-structured genotype, it is not more efficient to adjust for ancestry informative PCs. This is due to the very large (odds ratio > 10) association between the population and outcome and the exposure with a frequency of 50% (i.e., half the sample is in population 1 and the other is in population 2). When we extended Xing and Xing's simulations to larger exposure odds ratios with the exposure frequency of 50%, we obtained results consistent with our findings above. Whether to adjust for covariates depends on the complex relationships between the outcome, the covariate, and the genotype. When the phenotype does not have substantial structure, we obtain similar power when adjusting for population structure as when not adjusting for population structure. In their investigation, Xing and Xing limited to the situation where the covariate and genotype are independent. In our work, the SNP of interest and the ancestry informative PCs may not be independent. The SNP genotype is included in the linear combination of the genotypes that define the PCs; structured SNPs contribute a higher weight to some PCs. We found that with structured SNPs and non-structured phenotypes, it is more efficient to adjust for PCs.

For linear regression using continuous phenotypes, one can check phenotypes for association with the PCs. If a top PC is significantly associated with the phenotype of interest then the trait-genotype association model should include PCs as covariates to adjust for population structure. Unlike logistic regression, adjusting for covariates associated with the trait in linear regression always improves the precision of the effect estimate by reducing the residual variance [9]. Since the PCs are orthogonal, a single model regressing the top PCs on the outcome can be used to determine if the PCs are associated with the outcome. Associated PCs should be included as covariates in genome-wide association studies (GWAS).

Standard practice is to include a fixed number of PCs in association models for GWAS. Here, we conclude that if power is not a concern, then selecting the same set of PCs for adjustment for all SNPs in logistic regression is a strategy that achieves appropriate Type I error. However, standard practice is not optimal in all scenarios and to optimize power for structured SNPs in the presence of unstructured phenotypes, PCs that are associated with the tested SNP should be included in the logistic model. The gain in power we observed in our simulations was an approximate 5%-percentage point increase for adjusting only when the SNP is structured over always adjusting for the ancestry informative PCs. We note that some of the differences in power may disappear if we correct for Type I error, but this is not done in practice. It may be easier and more intuitive to adjusting for the same set of PCs across all SNP associations.

## Competing interests

The authors declare that they have no competing interests.

## Authors' contributions

GMP and KLL conceived and designed the study, carried out statistical analyses, and interpreted the data. GMP drafted the manuscript. KLL critically revised the manuscript. All authors read and approved the final manuscript.

## Supplementary Material

Additional file 1 **Supplemental Figure 1**. Empirical Type I error and power for increasing risk allele frequency differences. Click here for file

Additional file 2 **Supplemental Figure 2**. Empirical Type I error and power with 4 sub-populations. Click here for file

Additional file 3 **Supplemental Figure 3**. Power for 2 sub-populations and positive confounding. Click here for file
