# Supplementary material for: Choice of population structure informative principal components for adjustment in a case-control study
Source: BMC Genet. 2011 Jul 19;12:64. doi: 10.1186/1471-2156-12-64 (PMC3150322; doi:10.1186/1471-2156-12-64)

Supplemental Figure 1 - Empirical Type I error and power for increasing risk allele frequency differences

Two sub-populations of 500 individuals each, $F_{st} = 0.01$. p_1 is the risk allele frequency in sub-population 1 and p_2 is the risk allele frequency in sub-population 2. The x-axis is the various methods of selecting PCs for inclusion in the model of association and the symbols in the plot represent the phenotypic structure. The y-axis is the proportion of logistic regression models adjusting for the selected PCs for which the SNP p-values are significant at 0.05. The phenotypic ratio is the number of cases in sub-population 1 over the number of cases in sub-population 2.

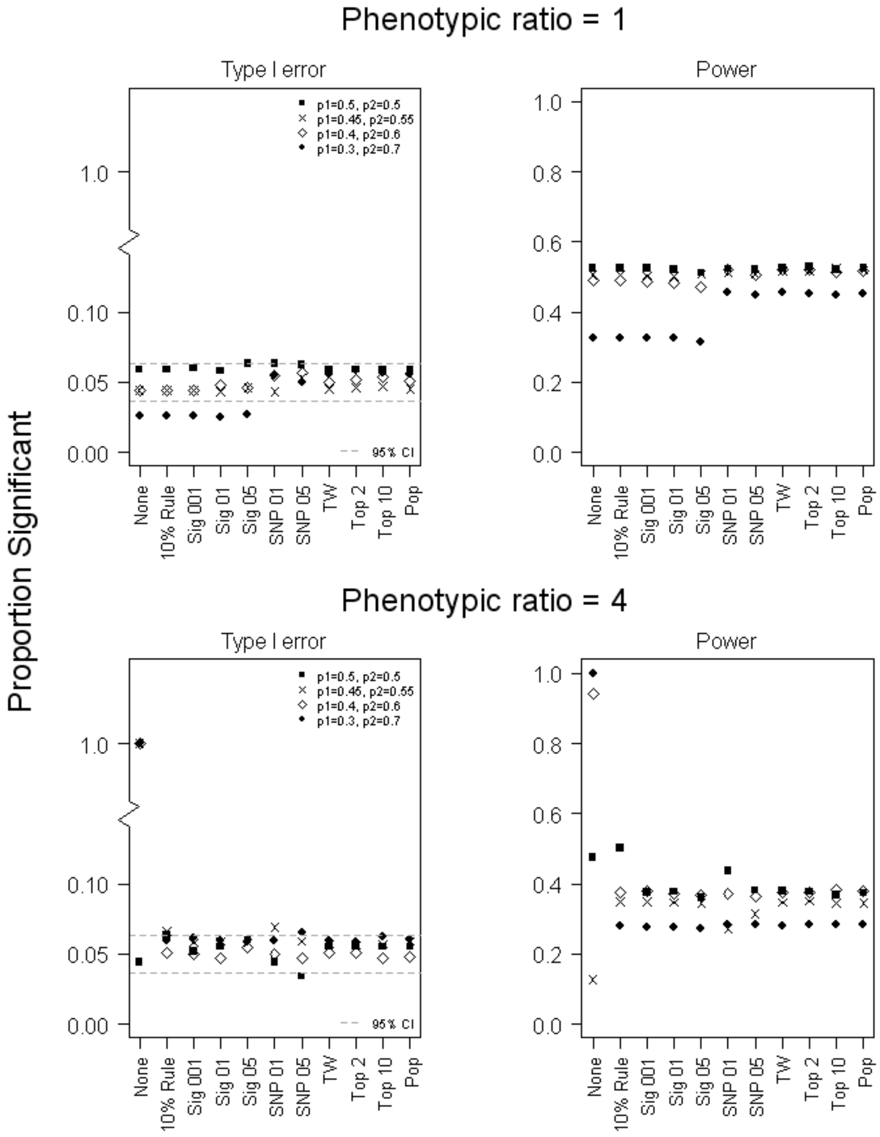

Supplement: Additional file 1 — Supplemental Figure 1. Empirical Type I error and power for increasing risk allele frequency differences. [file 1471-2156-12-64-S1.PDF]
