# Supplementary material for: Choice of population structure informative principal components for adjustment in a case-control study
Source: BMC Genet. 2011 Jul 19;12:64. doi: 10.1186/1471-2156-12-64 (PMC3150322; doi:10.1186/1471-2156-12-64)
Supplement: Additional file 2 — Supplemental Figure 2. Empirical Type I error and power with 4 sub-populations. [file 1471-2156-12-64-S2.PDF]

Supplemental Figure 2 - Empirical Type I error and power with 4 sub-populations

500 individuals in each sub-populations of 500 individuals, $F_{st} = 0.01$. The odds ratio for power was simulated to be 1.2 (log additive model). p_i is the risk allele frequency in sub-population i, i=1,2,3,4. The x-axis is the various methods of selecting PCs for inclusion in the model of association and the symbols in the plot represent the phenotypic structure. When the number of cases is the same in each of the sub-populations (125/125/125/125 = 125 cases from each sub-population), there is no phenotypic structure. When the number of cases is different in each of the sub-populations, there is phenotypic structure. The y-axis is the proportion of logistic regression models adjusting for the selected PCs for which the SNP p-values are significant at 0.05.

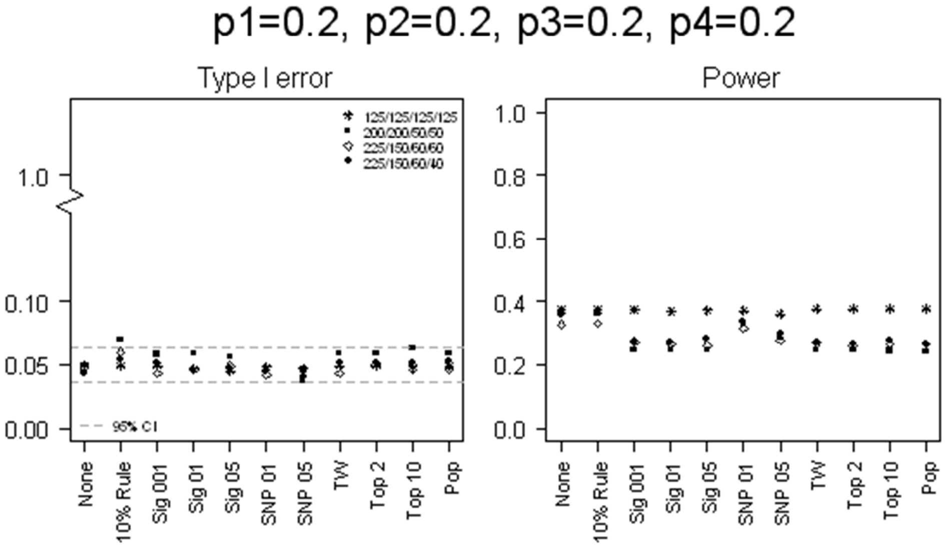

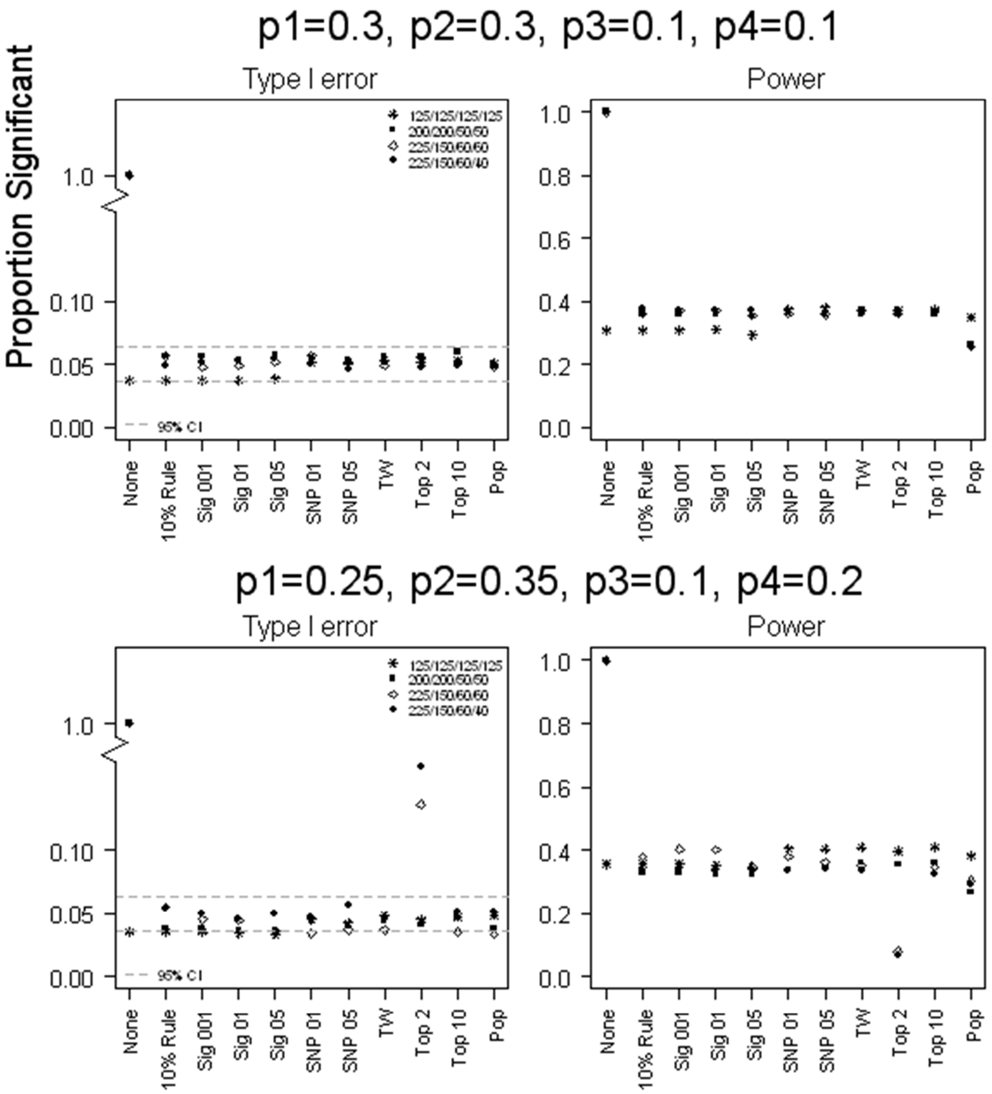
p1=0.3, p2=0.3, p3=0.1, p4=0.1
Proportion Significant
Type I error
Power
125/125/125/125
200/200/50/50
225/150/60/60
225/150/60/40
95% CI
1.0
0.10
0.05
0.00
0.8
0.6
0.4
0.2
0.0
None
10% Rule
Sig 001
Sig 01
Sig 05
SNP 01
SNP 05
TW
Top 2
Top 10
Pop
p1=0.25, p2=0.35, p3=0.1, p4=0.2
