# Supplementary material for: Choice of population structure informative principal components for adjustment in a case-control study
Source: BMC Genet. 2011 Jul 19;12:64. doi: 10.1186/1471-2156-12-64 (PMC3150322; doi:10.1186/1471-2156-12-64)

Supplemental Figure 3 - Power with 2 sub-populations and positive confounding

Two sub-populations of 500 individuals each, $F_{st} = 0.01$. K_1 is population prevalence of disease in sub-population 1 and K_2 is population prevalence of disease in sub-population 2. The risk allele frequency in population 1 was set to 0.3 and the risk allele frequency in population 2 was set to 0.1 to simulate positive confounding. Simulated log additive odds ratio of 1.2. The x-axis is the various methods of selecting PCs for inclusion in the model of association and the symbols in the plot represent the phenotypic structure. The y-axis is the proportion of logistic regression models adjusting for the selected PCs for which the SNP p-values are significant at 0.05.

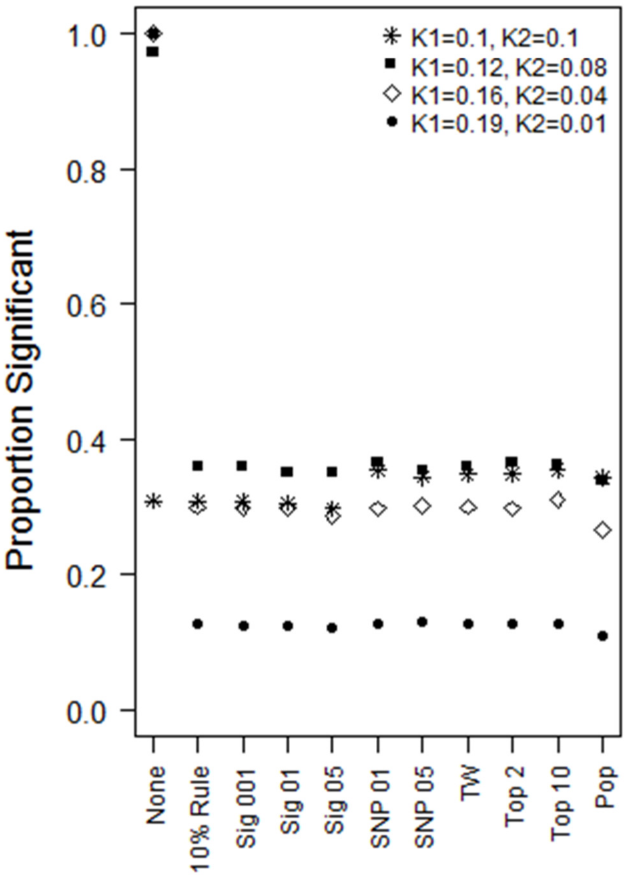

Supplement: Additional file 3 — Supplemental Figure 3. Power for 2 sub-populations and positive confounding. [file 1471-2156-12-64-S3.PDF]
